# The individual and societal burden of chronic pain in Europe: the case for strategic prioritisation and action to improve knowledge and availability of appropriate care

**DOI:** 10.1186/1471-2458-13-1229

**Published:** 2013-12-24

**Authors:** Harald Breivik, Elon Eisenberg, Tony O’Brien

**Affiliations:** 1 Department of Pain Management and Research, University Hospital and University of Oslo, Oslo, Norway; 2 Institute of Pain Medicine, Rambam Health Care Campus, Technion-Israel, Institute of Technology, Haifa, Israel; 3 Marymount University Hospice & Cork University Hospital, Cork, Ireland

**Keywords:** Chronic pain, Quality of life, Cost of illness, Pain management, Opioids, Pain burden, Drug toxicity

## Abstract

**Background:**

Chronic pain is common in Europe and elsewhere and its under treatment confers a substantial burden on individuals, employers, healthcare systems and society in general. Indeed, the personal and socioeconomic impact of chronic pain is as great as, or greater, than that of other established healthcare priorities. In light of review of recently published data confirming its clinical and socioeconomic impact, this paper argues that chronic pain should be ranked alongside other conditions of established priority in Europe. We outline strategies to help overcome barriers to effective pain care resulting in particular from deficiencies in education and access to interdisciplinary pain management services. We also address the confusion that exists between proper clinical and scientific uses of opioid medications and their potential for misuse and diversion, as reflected in international variations in the access to, and availability of, these agents.

**Discussion:**

As the economic costs are driven in part by the costs of lost productivity, absenteeism and early retirement, pain management should aim to fully rehabilitate patients, rather than merely to relieve pain. Accredited education of physicians and allied health professionals regarding state-of-the-art pain management is crucial. Some progress has been made in this area, but further provision and incentivization is required. We support a tiered approach to pain management, whereby patients with pain uncontrolled by non-specialists are able to consult a physician with a pain competency or a specialist in pain medicine, who in turn can recruit the services of other professionals on a case-by-case basis. A fully integrated interdisciplinary pain service should ideally be available to patients with refractory pain. Governments and healthcare systems should ensure that their policies on controlled medications are balanced, safeguarding public health without undue restrictions that compromise patient care, and that physician education programmes support these aims.

**Summary:**

Strategic prioritization and co-ordinated actions are required nationally and internationally to address the unacceptable and unnecessary burden of uncontrolled chronic pain that plagues European communities and economies. An appreciation of the ‘return on investment’ in pain management services will require policymakers to adopt a long-term, cross-budgetary approach.

## Background

Chronic pain is common in Europe and elsewhere and its under-treatment confers a substantial burden on individuals, employers, healthcare systems and society in general [1-4]. International resolutions have declared adequate pain therapy to be a human right [5-7] and chronic pain has been raised as a bioethical issue [8], and yet untreated chronic pain is under-recognized by health policymakers as a serious chronic health problem [5].

In this paper we argue that chronic pain should be ranked alongside other conditions of established priority in Europe, in light of recent data confirming its clinical and socioeconomic impact. We outline strategies, from a public health perspective, to help overcome barriers to effective pain care resulting in particular from deficiencies in education and access to interdisciplinary pain management services [9]. We also address the evident confusion that exists between the proper clinical and scientific uses of opioid medications and their potential for misuse and diversion, as reflected in international variations in the access to, and availability of, these agents. This paper is based on literature identified from the Pubmed database using various combinations of search terms appropriate to the aspect of pain medicine concerned, including ‘chronic pain’ , ‘cost of illness’ , ‘economic’ , ‘socioeconomic, ‘education’ , ‘opioids’ , ‘treatment’ , ‘multidisciplinary’. We prioritized papers according to their relevance and newness.

### Chronic pain is a leading health and socioeconomic problem

#### Chronic pain is common

In 2003, almost one in five surveyed Europeans reported having moderate or severe chronic pain, defined as pain lasting at least 6 months duration and with moderate to severe pain being experienced in the last month and at least twice a week [1]. This proportion varied from 12% in Spain to 30% in Norway. Almost 90% of individuals with chronic pain had experienced it for over 2 years and yet a third of sufferers were currently not being treated. The most common sites of pain were the back and joints, followed by head and neck pain, and common causes were spinal problems, pain after trauma and surgery [1]. More recent population-based surveys in various countries — including Spain [10], Portugal [11], Ireland [12], Denmark [13], Norway [14] and Iceland [15] — have consistently estimated that 25–35% of adults report chronic pain (Table 1). Notably, these studies did not use a common definition of chronic pain, such as that of the International Association for the Study of Pain [16]. An age-standardized analysis of 18 national surveys involving approximately 42,000 adults found that 37% of respondents in developed countries, and 41% in developing countries, reported a chronic pain condition [2]. In Europe, rates exceeded 40% in Italy, France and Ukraine. Differences between pain prevalence rates may partly reflect differences in the definitions of chronic pain used, in severity of pain included, and in selection of subjects (the most elderly and infirm persons, including those living in nursing homes, are often not included).

**Table 1:** Prevalence of chronic pain in epidemiological studies among adults in selected studies in Europe

**Country and survey year**	**Europe and Israel, 2003**^**1 **^[1]	**Denmark, 2010 **[13]	**Iceland, 2003 **[15]	**Ireland, 2008 **[12]	**Norway, 2006–2008 **[14]	**Portugal, 2007–8 **[11]
Survey method	Telephone	Postal or online	Postal	Postal	Postal	Telephone
Sample source and size	Telephone directories (n = 46,394)	National Danish Health Survey (n = 14,925)	National registry of general population (n = 599)	33 general practices (n = 1204)	Total regional adult population (n = 4782)	Telephone directories (n = 5094)
Chronic pain definition^2^	≥6 months duration, moderate or severe, and pain experienced in the last month and at least twice a week	≥6 months duration	>3 months duration	>3 months duration	Moderate to severe pain (SF-8) in at least three of five consecutive 3-monthly measurements	≥3 months duration
Prevalence of chronic pain (95% CI)	All: 19% (ND)	All: 26.8% (26.1–27.5%)	All: 30.6% (ND)	Non-cancer: 35.5% (32.8–38.2%)	31% (30–33%)	All: 36.7% (35.3–38.2%)
	(12% in Spain to 30% in Norway)	Non-cancer: 24.7% (ND)				
Prevalence higher in	Women, older age	Women, older age, various co-morbidities, non-Western background,^3^ underweight or obese	ND	Older age, manual workers, unemployed	Women, older age, lower educational level, lower household income, higher BMI	Women, older age retired, unemployed, lower educational level

BMI, body mass index; CI, confidence intervals; ND, no data; SF-8, Short-Form 8 health survey.

^1^Austria, Belgium, Denmark, Finland, France, Germany, Ireland, Israel, Italy, the Netherlands, Norway, Poland, Spain, Sweden, Switzerland and the UK.

^2^The International Association for the Study of Pain (IASP) defines chronic pain as that lasting more than 3 months [16].

^3^As determined by the birthplace and citizenship of the respondent and the parental birthplace.

Chronic pain is more common among manual workers and the unemployed than among professional workers [12], and more common among recipients of social assistance than the general population [17]. Chronic pain becomes more common with increasing age, especially in elderly women [1,2,11-14]. Elderly patients in pain are commonly undertreated or, more seriously, inappropriately treated in hospitals and nursing homes [18,19] — settings generally excluded from population-based surveys. In Italy, for example, pain was present in almost two thirds of patients (n = 367) in hospital geriatric departments; only 49% of these patients were receiving adequate pain therapy [18]. Across Europe, pain was reported in around third of nursing home residents [19] and inadequate pain control was the second most common deficiency identified in elderly home care [20]. The prevention and effective management of chronic pain in the elderly will be increasingly important as the population ages.

Half of all cancer patients, and up to 80% of those at advanced or terminal stages, experience chronic pain [13,21-24]. Around half of patients with cancer pain are undertreated [25,26]. Perhaps less well recognised are the high rates of chronic pain among people with other comorbid conditions. Indeed, new data suggest that pain may be at least as common in patients with cardiovascular disease, chronic pulmonary disease and chronic renal disease as in those with cancer [13,27].

### Chronic pain impairs quality of life, work and functioning

Chronic pain markedly decreases individuals’ health status and quality of life (QoL) [10,11,28-31] and can detrimentally affect the families of patients [32]. The degree of this effect is grossly underestimated. Patients admitted to a multidisciplinary pain centre in Norway reported health-related QoL as poor as that in patients with advanced cancer under palliative care [28]. Chronic pain is consistently linked with an increased risk of depression [10,12,33-35].

Chronic pain often interferes with everyday activities, such as family and home responsibilities, recreational activities (including exercise) and sleep [1,11,12,26]. At least half of people with chronic pain report that it interferes with their work [1,11,26]. Across Europe, almost one in five surveyed patients with chronic pain reported having lost their job because of their pain and one third reported that the hours they work, or whether they work at all, is affected by their pain [1]. Neuropathic pain may have a particularly severe impact on work [36]. Importantly, chronic pain can last many months, or indeed many years [1]. A recently published systematic review revealed that approximately two thirds of patients with non-specific lower back pain still experience pain 1 year after its onset, contrary to a commonly held belief that this normally resolves spontaneously in most patients [37]. In Finland, researchers found that chronic pain accounted for up to 30% of medically certified absence lasting more than 2 weeks [38] and that it was independently associated with early retirement due to disability [39]. Other studies have also documented the contribution of chronic pain to early retirement and disability pensions [40,41].

### Chronic pain: as costly as other prioritized diseases?

Recent studies [30,41-43] have confirmed previous evidence of the enormous indirect socioeconomic costs due to chronic pain [4,38,44-47]. In the US, the total costs associated with persistent pain in adults are now estimated at $560–635 billion (2010 prices) [42]. These costs are reported to exceed those estimated for heart disease ($309 billion), cancer ($243 billion) and diabetes ($188 billion) [42], although methodological differences limit the comparability of these values. In Europe, national healthcare and socioeconomic costs of conditions associated with chronic pain run into billions annually and represent 3–10% of gross domestic product (Table 2) [30,41,43]. Direct healthcare costs and indirect costs each account for approximately half of the total costs, with some international variation (Table 2). Hospitalization is the largest single component of direct costs, while social benefits (e.g. disability allowance and unemployment benefits) make the biggest single contribution to indirect costs [30,40]. In Sweden in 2008, for example, indirect costs of sick leave longer than 15 days and early retirement accounted for 59% of the total costs in patients with diagnoses related to chronic pain, followed by outpatient care and inpatient care [43].

**Table 2:** Recent studies on economic impact of chronic pain and conditions with which it is associated

**Country (pricing year)**	**Ireland (2008) **[30]	**Sweden (2008) **[43]	**Denmark (2010) **[41]	**United States (2010) **[42]
Data source	Postal survey	National and regional healthcare administrative registries	National administrative healthcare registries	Medical Expenditure Panel Survey
Pain definition	Chronic pain and conditions it	Diagnoses related to chronic pain	Pain-intensive diagnoses (n = 1,918,823)	Pain limiting ability to work; diagnoses of joint pain or arthritis; disability limiting ability to work (n = 20,214)
	features (n = 140)	(n = 837,896)		
Total cost/patient/year	€5,665	€6,429	Healthcare costs: DKK34,784–208,830/year (depending on condition), 2010	ND
Type of cost (% of total)	Direct healthcare: 52%	Direct healthcare: 41%	Direct healthcare: 71%	Direct healthcare: 47%
	Indirect: 48%	Indirect: 59%	Indirect: 29%	Indirect: 53%
National cost estimate/year	€5.34 billion	€32 billion	DKK17.8 billion	$560–635 billion
	~3% of GDP	~10% of GDP		~4% of GDP^a^

GDP, gross domestic product; PRIME, Prevalence, Impact and Cost of Chronic Pain; ND, no data.

^a^Not in original publication. Assumes US GDP in 2010 of US$14.4 trillion [48]).

**Figure 1:**
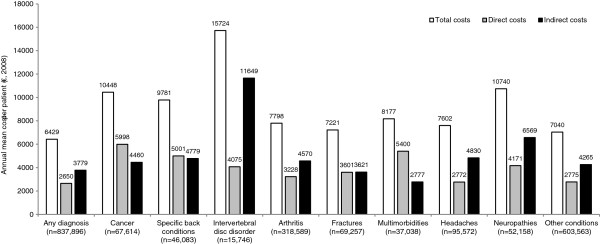
**Annual mean costs per patient in Sweden (2008) in patients with a diagnosis related to chronic pain, by diagnosis group and type of cost **[43]**.**

Among the types of pain, back pain conditions, cancer and neuropathies appear most costly (Figure 1) [43,47]. In Germany, societal costs of back pain have been estimated at €16.5–€50 billion when results from multiple studies are converted to 2008 prices. The bulk of this cost resulted from the impact of the condition on work [49]. In Portugal, the total indirect cost of chronic back and joint pain in 2008 was estimated at approximately €740 million, with productivity losses estimated at 0.5% of GDP [50].

Collectively, these data suggest that chronic pain states and the conditions with which they are associated present a burden at least as great, or perhaps even greater, than conditions that are conventionally prioritized as public health concerns. Productivity losses, absenteeism and early retirement and disability retirement contribute substantially to these costs, and would be best reduced by investment in improved diagnostic and therapeutic interventions that promote rehabilitation.

Importantly, these cost estimations are hampered by the difficulty in identifying patients with chronic pain within public health and health insurance registries. The International Classification of Diseases (ICD)-10 system does not have an adequate and distinct diagnostic code for chronic pain, although such a code has been created in Germany [51]. It is to be hoped that the ICD-11 system, presently under development, better represents chronic pain and thereby aids future research.

## Discussion

### How can the many problems regarding chronic pain be addressed?

The various attitudinal, educational, legislative, bureaucratic and economic barriers to effective pain management have been well documented by the World Medical Organization [6], the World Health Organization (WHO) and others [24,52]. We believe that at the root of these many problems are a lack of knowledge and awareness of the huge impact chronic pain has on quality of life of patients and on health care resources. Therefore, first we focus on strategies to tackle important deficiencies in knowledge and skills of pain management among healthcare professionals, in the provision of multidisciplinary care and in the availability and affordability of such care and medications.

### Education is key to improving knowledge of the burden of pain and its management

Pain management should include a thorough assessment of the type and severity of pain, the underlying causes, any associated co-morbidities or psychological problems, and — where necessary — an interdisciplinary therapeutic approach that aims both for pain relief and the restoration of physical, social and emotional functioning. Limitations in training among non-pain specialist physicians and other health professionals are an important barrier to improving patient care. For example, recent survey data confirm that European primary care physicians find chronic non-malignant pain a challenge to treat [53]. Key aspects of professional education include evidence-based guidelines and structured under- and post-graduate education.

#### Evidence-based guidelines for the management of chronic pain

The WHO three-step analgesic ladder for cancer pain relief has been widely influential [54]. The principle of offering appropriate analgesia in a timely fashion as articulated in the WHO ladder for cancer pain remains valid today, although the optimal approach remains a matter of debate and research [55]. The WHO has recently published guidelines for the management of persistent pain in children with medical illnesses [56] and is developing guidelines for non-malignant pain in adults [57]. Numerous other national and international guidelines for the pharmacological treatment of non-cancer and cancer pain exist [58-65]. While some guidelines for chronic non-cancer pain are evidence-based, for example those by Attal et al. on the pharmacological treatment of neuropathic pain [59], others are based primarily on expert opinion owing to a lack of well-designed, randomized and controlled trials in this area.

#### Impact of guidelines: do they make a difference?

Of course, the impact of guidelines is dependent on their implementation and levels of adherence among practitioners, and improving this is an important aim [66-69]. For example, evidence from the USA [68] and Europe [69] suggests that many primary care physicians are non-compliant with guidelines for chronic low back pain (LBP). Adherence to the WHO cancer pain guidelines should provide adequate pain control in the majority of patients [70,71], and yet pain remains common among cancer patients [25,26]. Researchers in Norway recently found that approximately 60% of persistent opioid users with chronic non-malignant pain receive concomitant regular benzodiazepines or benzodiazepine-related hypnotics, in conflict with guidelines [66].

Evidence from Germany suggests that an active approach to implementing LBP guidelines, using physician education interventions and motivational counselling may be more effective than simple postal dissemination of the guideline [72,73]. Certainly, guideline implementation and adherence among practitioners is likely to be aided by enhanced collaboration between professional societies and healthcare providers, policymakers, reimbursement authorities and health technology assessment authorities. However, further research is required to establish the optimal means of guideline implementation.

#### Pre- and post-graduate education in pain medicine for healthcare professionals

Substantial advances have been made in recent years in the scientific understanding of pain and its origins. One of the principal challenges in converting this progress into benefits to patients is the education of healthcare professionals regarding the optimal diagnosis and management of an increasingly complex variety of pain syndromes. All physicians should receive a basic education in pain management at undergraduate level, as recently re-iterated by the WHO in its 2011-updated recommendations on achieving balance in availability and treatment of pain with opioids [52]. In 2013, the European Federation of IASP Chapters (EFIC®) published its pain management core curriculum for European medical schools [74]. In reality, the provision of undergraduate pain education varies within and between countries and important deficiencies have recently been identified [75]. In part, this variation reflects international differences in the organization and governance of universities. In Germany, the content of medical education curricula is defined federally and a pain examination is now mandatory [76]. The provision of dedicated undergraduate pain modules is particularly common in France owing to a central policy [75]. However, standardized requirements are less feasible in countries where universities independently determine their own curricula (e.g. Austria, Norway and most Nordic countries, Spain, Italy, and Israel).

Specialized post-graduate education is also required to develop the expertise necessary to effectively manage patients with chronic pain. Two levels of post-graduate education can usefully be distinguished: 1) a diploma-based competency in pain management available to all types of physicians, and 2) accreditation of fully-fledged, cross-disciplinary pain medicine specialist qualification and role. Post-graduate pain management courses are now available in many countries [77]. In Italy, for example, a recent law (2010) means that physicians wanting to work in pain therapy can attain a specialist post-graduate Masters qualification in pain therapy or palliative care [Dr Massimo Allegri, personal communication]. These are likely to be particular useful for general practitioners, orthopaedic specialists and medical oncologists, the groups responsible for managing many patients with chronic pain [1,26]. In order to ensure high standards, pain medicine qualifications should preferably be regulated by regional or European-wide accreditation of courses, e.g. through the European Union of Medical Specialists (UEMS) [77], or EFIC. IASP and EFIC are already active in providing Pain Schools, e-learning resources [78], and grants to support education initiatives in Eastern Europe [79].

Pain medicine is now recognized as a speciality, sub-specialty or competency-based training in several European countries (e.g. Finland, Germany, Ireland, Israel, Norway, Sweden, UK and others). However, in many others advanced pain medicine as an area of modern medicine that requires special training and experience remains under-recognized among health managers and policymakers, and within the medical profession itself. This may be in part because the true burden of pain is still poorly documented, and because pain crosses so many fields of medicine. Only pain specialists have a patient-centred, multidisciplinary overview of all aspects of pain management. Pain specialists have important roles in the development and implementation of local, national and international guidelines, leading the development of pain care services, assessing and improving the value of pain care services through further research, advising governments and health authorities with regard to policy matters affecting pain (e.g. regarding access to controlled medicines), and leading public education. The pain field would also benefit from the development of best practices (e.g. protocols and policies) designed to raise standards of care. A good example is the guidelines on diagnosis and management of complex regional pain syndrome by the UK National Institute of Excellence (NICE) and Royal College of Physicians [80].

Pain education must also be properly covered within the under- and post-graduate education of other healthcare workers, including psychologists, nurses and pharmacists. In each case this requires interdisciplinary co-operation between pain societies, professional societies of allied healthcare professionals, educational institutions and healthcare systems.

#### Education of patients and the public

Various cognitive and educational barriers among patients may interfere with pain management by reducing adherence with treatment regimens [81,82]. Suggested components of public education include how to prevent common types of pain, how and when patients should self-treat pain, when patients should consult a doctor, what they can expect from therapy and how they can access further forms of support. From a public awareness perspective, it is important to stress that severe chronic pain is not acceptable and is not a part of normal ageing.

The provision of pain education for patients varies internationally. Efforts to improve this situation include an initiative to provide collaborative recommendations on improving education for older adults [83]. EFIC and IASP run annual Year Against Pain events in an effort to increase public awareness of pain issues in the public and media, as well as the medical community (http://www.efic.org/index.asp?sub=F8AMLHLAP9216P).

Patient education or coaching measures helped to improve pain, functioning, well-being and therapy adherence in some studies in patients with LBP [84,85] and cancer pain [86-88]. However, a recent systematic review concluded that the available data on education interventions in LBP were of low quality and showed no intermediate- or long-term effect on pain and disability compared with active non-educational interventions [89]. Mass media campaigns based on education alone are unlikely to result in positive and persisting behavioural change and need to be supported by other approaches, for example based on social marketing, policy and legislation [90]. In Norway, a mass media campaign on LBP (involving written educational materials sent to all households, television, radio and cinema advertisements and posters in health clinics) had only a limited effect on the beliefs of survey respondents among the general public, as compared with controls not exposed to the campaign [67,91]. Furthermore, it did not significantly affect sickness behaviour (i.e. sickness absence, surgery rates for intervertebral disc herniation and imaging examinations) or change the beliefs of physicians, physiotherapists and chiropractors regarding LBP, even though an additional educational initiative was directed at these groups [92]. These results suggest that a considerable investment would be needed to improve public understanding and behaviour with regard to chronic pain.

### How can pain management services be optimized?

#### Interdisciplinary pain management

Ideally, patients with uncontrolled chronic pain should have access to a full range of diagnostic and therapeutic modalities appropriate to their case, which may include pharmacological therapy, physiotherapy, clinical psychology, surgery, invasive techniques, occupational therapy and rehabilitation medicine. Current data suggest that access to integrated interdisciplinary pain management varies across Europe. In Norway, for example, patients now have a legal right to receive prioritized healthcare in multidisciplinary pain clinics if their health-related QoL is severely affected by the pain condition and efficacious and cost-effective treatment is available [93]. In Italy, a law passed in 2010 defined patients’ rights to access to multidisciplinary pain centres and promoted the development of regional and national networks of centres and care pathways [94,95]. In contrast, patients in many countries have limited access to these services, and even where multidisciplinary pain centres do exist there can be prolonged waiting times.

Evidence suggests that adding additional interventions such as advice, education, exercise, rehabilitation or occupational therapy to usual care provided by general practitioners is cost-effective from a societal perspective, i.e. when loss of earnings and productivity losses are taken into account [96,97]. Comprehensive multidisciplinary assessment and management programmes are costly and there have been few well-designed evaluations with long-term follow-up. The available studies are heterogeneous, of variable quality, and offer only limited evidence for an intermediate or long-term benefit or for cost-effectiveness [89,98-100]. Positive reports from Denmark [99,101,102] and Sweden [96] on a multi-professional approach to chronic non-cancer pain are supported by outcome data from a programme in Germany (preliminary data published in non-peer reviewed articles in the German language) [103,104]. In Finland, 46% of 439 patients had improved QoL after 6 months’ treatment in a multidisciplinary pain clinic, and benefits were shown to last for at least 3 years [105,106]. A randomized 12-month study in Spain showed significant benefits of multidisciplinary care in patients with fibromyalgia [107].

Thus, although there is considerable support for the cost-effectiveness of interdisciplinary management of complex chronic pain conditions, there is a pressing need for large, well-designed randomized trials to further evaluate the effectiveness of chronic pain interventions and their cost-effectiveness from a societal perspective, as well as a cost-benefit from the perspective of healthcare providers and patients. The initiation and funding of projects such as the European Commission-funded PAIN-OUT project (http://www.pain-out.eu) is also recommended. This project aims to develop and validate a system for the continuous measurement, feedback and benchmarking of treatment quality in postoperative pain, and to reduce the risk of chronic postoperative pain with the incorporated European Society of Anaesthesiologists project on chronic postsurgical pain (euCPSP).

#### Organizing pain management: from general practice to specialized pain clinics

It is unrealistic to suggest that all patients with chronic pain should routinely be seen by numerous different health professionals. Rather, we would define a tiered approach to pain management that could be applied, with adaptations, in most countries.

Firstly, *general practitioner* (GP) or non-specialist physicians will have to manage most patients with chronic pain, and hence improvements in the basic pain education of this group of doctors are essential. Guidelines for pain management by GPs are important, but helpful only if known and accepted by the GPs [68,69]. Secondly, patients whose pain is not controlled by these professionals should be referred to an *organ or disease specialist* according to the aetiology of the pain, i.e. to an orthopaedic surgeon in the case of hip pain, a neurologist in the case of headache, and so forth. Referral to physician with a *special pain competency* achieved through a post-graduate qualification, or to *a registered pain specialist or subspecialist*, is necessary when a cause is identified which cannot be reversed by specific medical or surgical treatment and/or if standard measures prove ineffective. These physicians should be able to perform a more detailed, tailored evaluation, to request assistance from other professionals as appropriate, and to undertake close monitoring of patients with the adjustment of therapy as appropriate. *Interdisciplinary pain centres* represent the most specialized tier of care, where a range of professionals with expertise in pain management collaborate in a fully integrated, state-of-the-art service [108]. Importantly, many patients with chronic pain do not consult a doctor at all [1]. The reasons for this are unclear and efforts are needed to improve appropriate healthcare-seeking behaviour among pain sufferers.

*Healthcare decision-makers* may under-estimate the benefits of an interdisciplinary approach to pain management, or to view them as too nebulous, diffuse, costly and ineffective. This could result from the lack of robust data quantifying the burden of pain in relation to other health problems, and supporting the cost-effectiveness of physicians with special competence in pain medicine, as well as interdisciplinary care. Recent studies discussed above have provided some insight, but further research is required.

#### The importance of savings in social care costs and societal budgets from pain management programmes

Importantly, the managerial separation (‘ring-fencing’) of healthcare and social care budgets hinders an appreciation of how, with pain management programmes, the former are anticipated to be offset by savings in the latter. A cross-budget approach is therefore required when evaluating the case for investing in improved pain management. Furthermore a long-term perspective is needed owing to the lag between investment in pain management and prevention and reduced social care costs [96,102]. Crucially, research is needed to quantify the effects of improved pain management on absenteeism, presenteeism, employment and social care costs. In Norway, chronic pain is the direct cause of half of the cases of early or disability retirement [14,40]. This means that effective treatment and rehabilitation back to work in only a few of these cases could save a lot of costs in the social care budgets [40].

### Availability of controlled medications for pain: is there a problem?

Indeed, there is a problem — there being too much usage of these medications for a few patients, and too little use (or a lack of availability) for most people in the world [52].

Pharmacotherapy remains a cornerstone of pain management, and care quality is compromized if patients lack access or affordability to recommended prescribed medications. The availability of medications is limited by factors such as cost, licensing, prescribing regulations and cultural factors. These conditions vary widely between countries, even in Western Europe. Whereas Austria and north-western European countries have opioid usage that is second only to the USA and Canada, an extremely low-use of weak opioids and close to none-use of potent opioids for chronic non-cancer pain have recently been documented by an excellent epidemiological study in Portugal [109].

Issues relating to the availability of opioid analgesics, in particular, have been contentious for many years. In many countries — especially in Eastern Europe and in the developing world — access to opioid therapy remains inadequate owing to barriers such as limited subsidy, limited availability, and restrictive regulations on prescribing and dispensing [24,52,110-113].

### Availability and appropriate use of opioids for palliative care as well as for chronic non-cancer pain

Opioids are the mainstay of treatment of advanced cancer pain [54,61,63,64]. They also have a role in selected patients with chronic, moderate to severe non-cancer pain, although the evidence base for the efficacy and safety of long-term opioid therapy is limited [114-116] and additional well-designed studies are required [117-121]. The only published double-blind, randomized study of an opioid compared with placebo that lasted as long as 6 months revealed many of the difficulties in performing blinded, long-term opioid trials, namely those of: maintaining blinding in the presence of typical opioid effects and side effects, early dropouts in the placebo arm, and potent and persistent context-sensitive effects of close patient monitoring by an enthusiastic research pain team [115].

Precautions are necessary to minimize the risks of adverse events, misuse, dependence and misdirection when opioids are prescribed. These include careful patient selection with risk assessment, trial therapy periods and careful monitoring of patients [60,65]. Available trial data suggest that opioid abuse and addiction are rare during clinical therapy [120,122]. Nationwide registry data from Norway suggest that persistent or problematic use respectively occurred in 0.3% and 0.08% of patients prescribed weak opioids (e.g. codeine, tramadol and dextropropoxyphene) for non-cancer pain in clinical practice [123]. Approximately 0.16% of the general population persistently use strong opioids on prescription, but true “addiction” and abnormal drug-seeking behaviour are estimated to represent a small fraction of this percentage [124]. Further observational research on such outcomes during therapy with strong opioids used according to best practice would be helpful [122].

National policies controlling access to opioids differ widely internationally [125]. Health policymakers tend to be wary of increasing access to opioids because of fears and confusion about misuse, misdirection, addiction and tolerance. In the US, there are concerns that a large increase in opioid use over the last two decades has been damaging to public health [126]. However, these fears should not result in reduced access to appropriate therapeutic use with careful patient selection and supervision. The authors urge healthcare decision-makers at all levels to adopt a balanced approach between ensuring the availability of controlled medications for legitimate medical and scientific purposes while preventing their diversion and abuse, in line with that advocated by the WHO [52] and the International Narcotics Control Board [127]. Recent legislation in Italy has simplified the prescribing of opioids [93,94]. Significant progress has also been made in some Eastern European countries, e.g. Romania and Serbia, in replacing restrictive legislation, improving access and instituting appropriate medical education [128,129].

Some physicians lack confidence in prescribing opioids, especially for chronic non-malignant pain [130,131]. Evidence suggests that that education programmes can help to improve this [132], but changing pain treatment patterns is challenging [133,134]. Ultimately, prescribing is not the main difficulty in opioid therapy. Rather, it is essential that physicians undertake comprehensive patient and pain assessment and institute a trial of opioids using pre-determined outcome measures (e.g. a documented significant reduction in pain scores and evidence of improved physical and psychological functioning). Patients treated with opioids require careful monitoring, including assessments of early signs of misuse [135]. In light of this, it has been proposed that opioid therapy for non-cancer pain should be in the hands of specialists only, not in the hands of general practitioners. However, this would be difficult to enforce; improved knowledge among GPs and early referral of “complex” pain patients to pain specialists may help with this difficult dilemma. Instead of using a purely pharmacological focus, under- and post-graduate education on opioids must provide up-to-date instruction on the proper use of these agents in clinical practice.

### Availability and appropriate use of antihyperalgesic medication

Opioids are not the only analgesic medications that are often underused. Antihyperalgesic medications for chronic pain conditions with neuropathic components (e.g. gabapentin and pregabalin and some antidepressants) are often unavailable or unaffordable. Even when available, these agents are often not used, or are used inappropriately owing to a lack of knowledge of their recommended place in therapy and how to follow-up the gradual onset of their therapeutic and adverse effects [59]. Medical education must therefore cover the range of agents used in modern pain management and restrictions on the availability of all relevant agents should be subject to the balanced approach discussed above. Patients must also be informed regarding such aspects as the anticipated time-course of therapeutic benefits and adverse effects to help ensure that these agents are used optimally.

## Summary

Strategic prioritization and co-ordinated actions are required at the national and international levels to address the unacceptable and unnecessary burden of uncontrolled chronic pain that plagues European communities and economies. The personal and socioeconomic impact of chronic pain is as great, or greater than, that of established healthcare priorities such as cardiovascular disease and cancer. As the economic costs are driven in part by the costs of lost productivity, absenteeism and early retirement, pain management should aim to fully rehabilitate patients, rather than merely to relieve pain. Chronic pain must be recognized as a complex somatic and psychosocial disease state (rather than a solely a symptom) both to allow its epidemiology and impact to be better quantified and to drive improvements in care. Accredited education of physicians and allied health professionals regarding state-of-the-art pain management is crucial. Some progress has been made in this area, especially with the availability of post-graduate education in many countries, but further provision and incentivization is required. We support a tiered approach to pain management, whereby patients with pain uncontrolled by non-specialists are able to consult a physician with a pain competency or a specialist in pain medicine, who in turn can recruit the services of other professionals on a case-by-case basis. A fully integrated multidisciplinary pain service should ideally be available to patients with refractory pain, for example in ‘centres of excellence’. Governments and healthcare systems should ensure that their policies on controlled medications are balanced, safeguarding public health without undue restrictions that compromize patient care, and that physician education programmes support these aims. An appreciation of the ‘return on investment’ in pain management services will require policymakers to adopt a long-term, cross-budgetary approach.

## Competing interests

OPEN Minds (http://www.paineurope.com/topics/openminds) is a group of European experts specialising in the research and management of pain. It is funded by an educational grant from Mundipharma International Ltd (Cambridge, UK). The sponsor made no intellectual or editorial contribution to the content of this paper. HB has received honoraria from lectures, clinical research projects, advisory board participation, and the like, from a number of pharmaceutical companies marketing drugs in drug-classes mentioned in this paper: Pfizer, Mundipharma, Gruenethal, Wyeth, Weifa, Bristol-Myers Squibb, ProStrakan, Orion, Nycomed, Jansen, GlaxoSmithKline, Fresenius Kabi, AstraZeneca, Boehring Ingelheim, Astellas, Actavis. He does not have any stocks or shares in any companies. He does not have any patents, and is not applying for patents related to the contents of this paper. He does not have any nonfinancial competing interest related to the contents of this paper. EE has received research support from government and industry sources at various times, and consulted for and received lecture fees from various pharmaceutical companies related to analgesics and other healthcare interventions. TOB has received honoraria/financial support from various pharmaceutical companies arising from his work as an advisory board member, researcher, guest lecturer and consultant. TOB confirms that he does not hold any relevant stocks or shares and does not hold any patents.

## Authors’ contributions

All authors participated in a series of OPENMinds round-table meetings at which the scope and content of this article was discussed. All authors reviewed the manuscript at every stage of the drafting process, contributing intellectual content, editorial input and critical revision. All authors gave final approval for the publication of this article.

## Pre-publication history

The pre-publication history for this paper can be accessed here:

http://www.biomedcentral.com/1471-2458/13/1229/prepub
